# Bimanual coupling effect during a proprioceptive stimulation

**DOI:** 10.1038/s41598-021-94569-8

**Published:** 2021-07-22

**Authors:** M. Biggio, A. Bisio, F. Garbarini, Marco Bove

**Affiliations:** 1 Department of Experimental Medicine, Section of Human Physiology and Centro Polifunzionale di Scienze Motorie, University of Genoa, Viale Benedetto XV 3, 16132 Genoa, Italy; 2 MANIBUS Lab, Department of Psychology, University of Torino, Turin, Italy; 3 IRCCS Ospedale Policlinico San Martino, Largo Rosanna Benzi, 10, 16132 Genoa, Italy

**Keywords:** Cognitive neuroscience, Motor control, Sensorimotor processing

## Abstract

Circle-line drawing paradigm is used to study bimanual coupling. In the standard paradigm, subjects are asked to draw circles with one hand and lines with the other hand; the influence of the concomitant tasks results in two “elliptical” figures. Here we tested whether proprioceptive information evoked by muscle vibration inducing a proprioceptive illusion (PI) of movement at central level, was able to affect the contralateral hand drawing circles or lines. A multisite 80 Hz-muscle vibration paradigm was used to induce the illusion of circle- and line-drawing on the right hand of 15 healthy participants. During muscle vibration, subjects had to draw a congruent or an incongruent figure with the left hand. The ovalization induced by PI was compared with Real and Motor Imagery conditions, which already have proved to induce bimanual coupling. We showed that the ovalization of a perceived circle over a line drawing during PI was comparable to that observed in Real and Motor Imagery condition. This finding indicates that PI can induce bimanual coupling, and proprioceptive information can influence the motor programs of the contralateral hand.

## Introduction

Activity requiring a coordinated use of both hands simultaneously are frequent during our daily life. Even if the ability of performing very different movements in parallel with our limbs can be trained, such as musicians do, coordination often requires that two movements have the same spatial or temporal characteristics^1^. Doing two incompatible tasks at once often results in interference at the behavioural level, reflecting the central nervous system’s limitations in controlling different streams of action in parallel^2,3^.

Behavioural interference has been widely explored through the circle-line drawing task, a paradigm of bimanual coupling that requires to draw continuous circles with one hand and continuous lines with the other^4^. Despite these sketches are easy when executed unimanually, when performed together they tend to influence each other, resulting in two “elliptical” figures. This paradigm has been investigated in several conditions to answer which process underlies bimanual interference. In particular, bimanual coupling studies were performed in healthy people^5–7^, patients with sensory disturbance^8^, subjects not able to move^9,10^, phantom limb experience following upper limb amputation^11^, issue in sense of agency and movement representation^12–14^. Until motor intention is spared, effects of interference during bimanual tasks can be observed even without performing real movements. For example, Garbarini and colleagues showed in anosognosic patients (i.e., denial of paralysis^15^) a behavioural interference in a line-circle paradigm comparable to controls, whilst patients with motor neglect (with spared motor execution but damaged motor intention) did not show any coupling effect^13^. Further, the drawing of a figure is modified while participants imagined drawing with the other hand^3,13^.

Muscle vibration can selectively activate muscle spindle receptor, thereby inducing, at a vibration frequency ranging between 80 and 100 Hz, an excitation of the primary endings and a train of action potentials in the large diameter afferent fibres^16,17^. Further, at central level, muscle vibration can induce a vivid illusory sensation of movement dealing with the lengthening illusion of the vibrated muscle^17,18^. It must be noticed that the Proprioceptive Illusion (PI) emerging from tendon vibration is not just a simple peripheral stimulation with a sensory feedback. It has been argued that the illusion evoked by tendon vibration mimics the characteristics of the movement, rather than just inducing a mere sensation of muscle lengthening. According to Cordo^19^, movement illusions adapt even though the primary afferents responsible for these illusions continue to discharge at a constant rate. In fact, even if the mechanical stimulation does not change, the experienced movement has a defined onset and changes in movement’s velocity are perceived^19^.

The illusory sensation is mediated by an ensemble of sensorimotor and associative cortical and subcortical regions that partially overlaps the cortical network involved in motor planning and actual movement execution^20–26^, as for instance primary sensorimotor areas including the primary motor cortex and primary somatosensory cortex, somatosensory association cortex, supplementary motor area^20,27^. Most of those areas are highlighted by the neural cross-talk approach, one of the two frameworks attempting at explain intermanual transfer whereby the movement commands assigned to one hand spread to the neural centers controlling the other hand^1,8^.

A research carried on by our group showed that short-term upper limb immobilization induces a hemispheric unbalance between the primary motor cortices^28^. Also, we found that the maintenance of dynamic proprioceptive inputs in an immobilized arm, by means of muscle vibration, can prevent the hemispheric unbalance induced by short-term limb disuse^29^.

However, it is not clear if this effect is due to the localized proprioceptive integration in the sensorimotor areas of the stimulated limb or due to an amount of proprioceptive information actually transferred to the non-stimulated hemisphere. Therefore, bimanual coupling could be a useful tool to investigate the occurrence of proprioceptive information interhemispheric transfer during muscle vibration. It has been proposed that bimanual spatial and temporal motor constraints are tightly linked to abstract representations of action, rather than to movement execution. The results of studies involving healthy subjects have suggested that the interference effect cannot be modulated by manipulating afferent sources of information, concluding that spatial interference primarily emerges at the efferent level of movement planning and organization^8,30–32^. However, since we assume that PI is more than just an afferent input coding a muscle lengthening sensation, in this work we aim to demonstrate that the sensorimotor pattern evoked by muscle vibration could be transferred from one hemisphere to the contralateral one, influencing the motor program of the drawing task performed with the not stimulated hand. Specifically, 15 healthy participants were enrolled to perform a circle-line paradigm in a real (RE), motor imagery (MI) and proprioceptive illusion condition. In the latter condition, we induced the illusory sensation of drawing with their right hand lines or circles, testing the influence of the illusion over their left hand. The occurrence of the illusory movement perception in all subjects was preliminarily assessed. In order to evaluate the bimanual coupling effect, we computed the ovalization index (OI) that allowed quantifying the deviation from a perfect line or a perfect circle in both congruent (when the participants performed/imagined/perceived an illusory movement on the right hand similar to that performed with the left hand, namely circle-circle or line-line) and incongruent (when the participants performed/imagined/perceived an illusory movement on the right hand different from that performed with the left hand, namely circle-line or line-circle) conditions.

## Results

### Vividness

The results of the paired t-tests preliminary comparing the vividness of the illusion perceived by subjects of line’s and circle’ pattern showed no significant difference (Line vs. Circle: 7.38 ± 0.39 vs. 6.77 ± 0.23, *p* = 0.09). This means that, when subjects focus only on the vibration, the intensities of the illusion evoked by the two stimulation’s pattern were comparable.

### Ovalization

**Figure 1:**
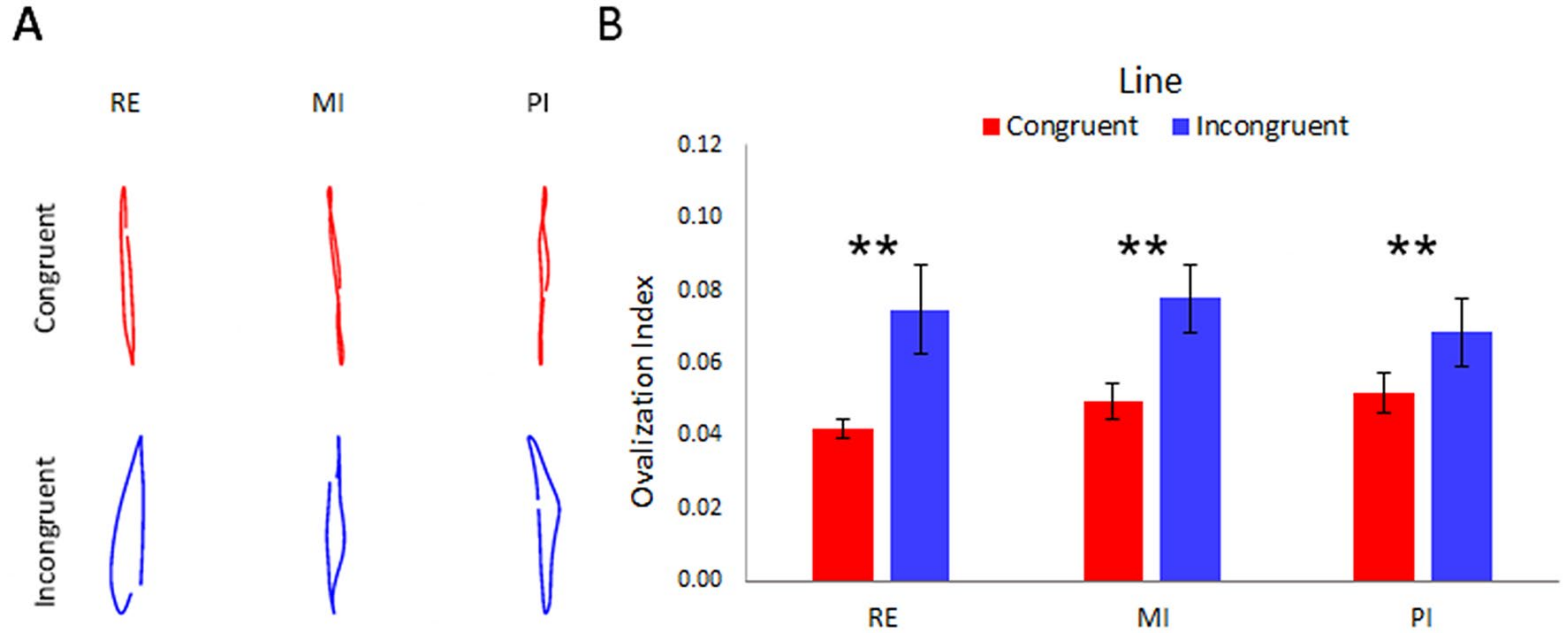
(**A**) Shows examples of a representative subject's left-hand trajectory in the three protocols (Real - RE, Motor Imagery—MI and Proprioceptive Illusion—PI). Red lines represent the congruent condition (namely, when the subject was performing/imagining/perceiving lines with their right hand, too). Blue lines represent the incongruent condition (namely, when the subject was performing/imagining/perceiving circles with their right hand). The averaged Ovalization Indexes of the line-drawings performed with the left hand in RE, MI and PI protocols during congruent (red bar) and incongruent (blue bar) conditions are represented in (**B**). The error bars refer to the standard error of the mean. **Refers to t-test results of corrected p < 0.016.

Figure 1 showed data related to the line drawing ovalization. The three conditioning protocols, in the Incongruent condition, induced a similar pattern of ovalization; namely, we observed a significant main effect for the factor CONGRUENCY (F_(1–14)_ = 20.18; *p* < 0.01;* ƞ*^2^ = 0.93), with the Incongruent condition showing a greater ovalization (OI—mean ± ES: 0.074 ± 0.006) than the Congruent condition (OI—mean ± ES: 0.048 ± 0.003). No significant differences were found between the three conditioning protocols (p = 0.76; *ƞ*^2^ = 0.21). In order to deeply investigate whether each protocol was effective to induce ovalization, we compared Congruent and Incongruent condition of RE, MI and PI with a paired t-test. We adjusted threshold levels of significance for multiple comparisons with Bonferroni correction, which resulted in a α = 0.017.

Results showed that the ovalization was significantly higher in RE Line-Incongruent that in RE_Line-Congruent (OI—mean ± ES: 0.075 ± 0.012 vs 0.042 ± 0.002, p = 0.001; *ƞ*^2^ = 0.76) and in MI Line-Incongruent that in MI_Line-Congruent (OI—mean ± ES: 0.078 ± 0.009 vs 0.050 ± 0.006, p = 0.006; *ƞ*^2^ = 0.43). Particularly, t-test comparing PI conditions showed significantly higher ovalization in PI Line-Incongruent that in PI_Line-Congruent (OI—mean ± ES: 0.068 ± 0.009 vs 0.052 ± 0.005, p = 0.016; *ƞ*^2^ = 0.41).

**Figure 2:**
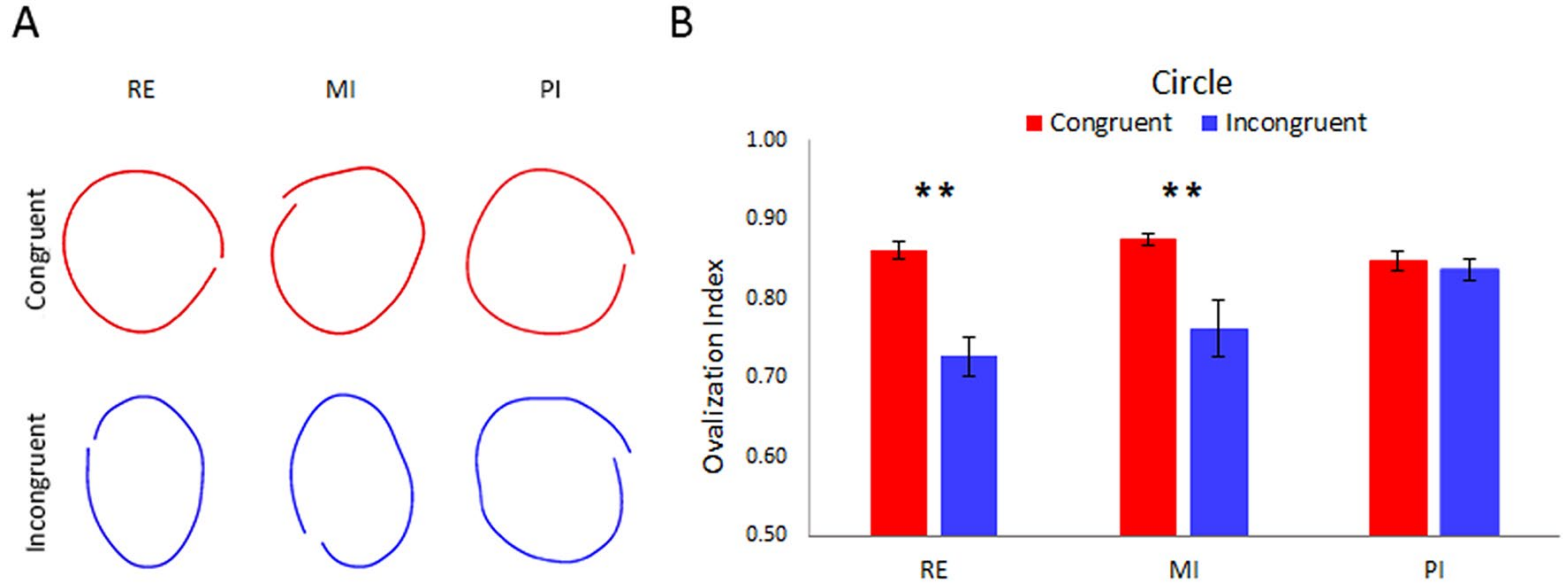
(**A**) Shows examples of a representative subject's left-hand trajectory in the three protocols (Real - RE, Motor Imagery—MI and Proprioceptive Illusion—PI). Red circles represent the congruent condition (namely, when subject was performing/imagining/perceiving circles with their right hand, too). Blue circles represent the incongruent condition (namely, when subject was performing/imagining/perceiving lines with their right hand). It is noticeable the presence of ovalization in the incongruent condition in the RE and MI protocol but not in PI. The averaged Ovalization Indexes of the circle-drawings performed with the left hand in Real, MI and PI protocols during congruent (red bar) and incongruent (blue bar) conditions are represented in (**B**). The error bars refer to the standard error of the mean. *Refers to p < 0.05.

Circle task’s data are shown in Fig. 2. Here the RE and MI conditioning protocols showed a greater ovalization of the Circle during the Incongruent condition than the PI protocol.

Indeed, ANOVA showed a significant main effect of the factors PROTOCOL (F_(1–14)_ = 4.17, *p* < 0.05;* ƞ*^2^ = 0.81) and CONGRUENCY (F_(1–14)_ = 38.12, *p* < 0.01;* ƞ*^2^ = 0.79).

Further, a significant interaction between PROTOCOL and CONGRUENCY factors was found (F_(2–28)_ = 7.72, p < 0.01;* ƞ*^2^ = 0.88). Post hoc analysis showed a significantly higher ovalization in Incongruent than Congruent condition in Real and MI protocols (always p < 0.01), with no significant differences between these two (RE vs. MI were comparable in both conditions). Data are summarized in Table 1.

**Table 1:** Mean values and standard error of the ovalization indexes of lines and circles in Real (RE), Motor Imagery (MI) and Proprioceptive Illusion (PI) protocols and in Congruent and Incongruent conditions.

Ovalization index			
	RE	MI	PI
**Line**			
Congruent	0.04 ± 0.00	0.05 ± 0.01	0.05 ± 0.01
Incongruent	0.07 ± 0.01	0.08 ± 0.01	0.07 ± 0.01
**Circle**			
Congruent	0.86 ± 0.01	0.87 ± 0.01	0.85 ± 0.01
Incongruent	0.73 ± 0.02	0.76 ± 0.04	0.84 ± 0.01

### Mean velocity

Mean velocity for Lines and Circles showed similar behaviors.

For Lines, ANOVA showed a significant main effect of PROTOCOL factor (F_(1–14)_ = 3.75, *p* < 0.05; *ƞ*^2^ = 0.21). Post-hoc analysis revealed that in RE protocol subjects were faster than in the others (RE vs. MI: 90.21 ± 10.71 mm/s vs. 72.89 ± 8.59 mm/s, *p* < 0.05; RE vs. PI: vs. 69.49 ± 7.62 mm/s, *p* < 0.05).

**Figure 3:**
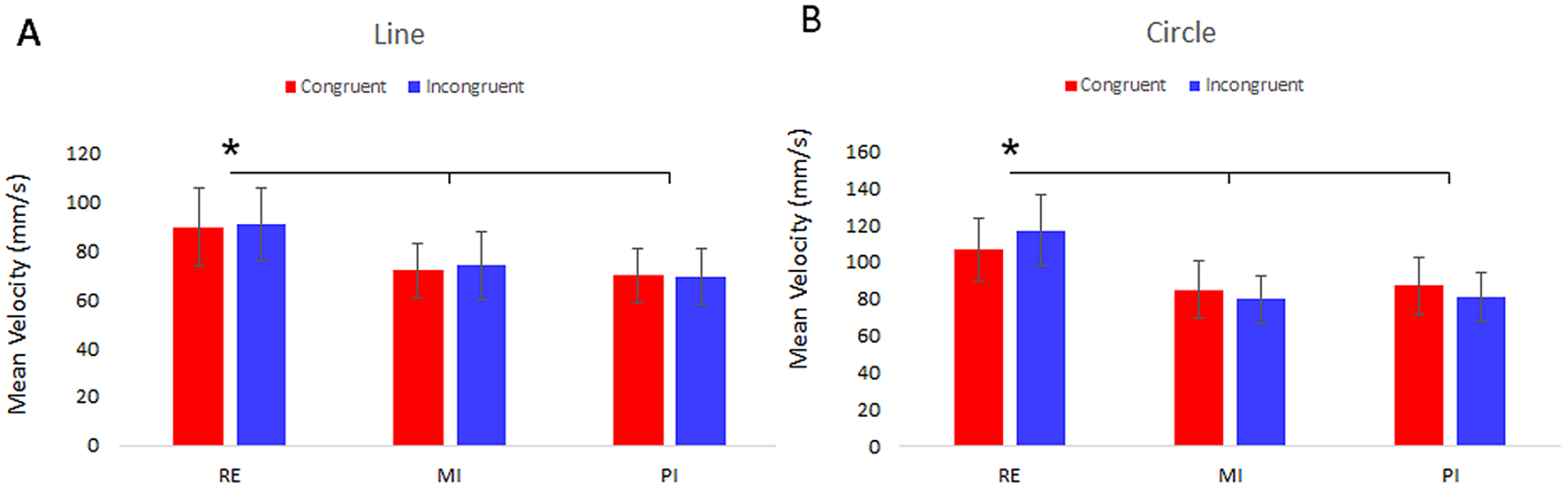
(**A**) Shows averaged Mean Velocity of the line-drawings performed with the left hand in Real, MI and PI protocols in Congruent (red) and Incongruent (blue) conditions. (**B**) Shows averaged Mean Velocity of the circle-drawings in the three protocols and in the Congruent and Incongruent condition. The error bars refer to the standard error of the mean. *Refers to p < 0.05.

For circles, ANOVA showed a significant main effect of PROTOCOL factor (F_(1–14)_ = 5.86, *p* < 0.05;* ƞ*^2^ = 0.29). Post-hoc analysis revealed that in RE protocol subjects were faster than in the other conditions (RE vs. MI: 111.85 ± 12.53 mm/s vs. 82.47 ± 9.82 mm/s, *p* < 0.05; RE vs. PI: vs. 84.12 ± 9.94 mm/s, *p* < 0.01). Mean velocities of Lines and Circles in the three protocols are shown in Fig. 3.

## Discussion

In this work we assessed whether the illusion of movement evoked by muscle vibration could induce bimanual interference on the contralateral hand performing an incongruent concurrent task. To this aim, a circle-line task, known to detect bimanual coupling, was employed in real, motor imagery and proprioceptive illusion conditions. We compared the ovalization in the trajectory of the drawing actually performed with the left hand while performing/imagining to perform/perceiving the illusion of drawing lines or circles with the right hand.

We found that the lines drawn by participants while perceiving with the other hand the illusion to draw circles were more ovalized than those during the congruent illusion. Moreover, the ovalization of the lines in the incongruent conditions was comparable for Real, Motor Imagery and Proprioceptive Illusion protocols. This finding points out that bimanual coupling occurred during PI, in line with previous studies investigating bimanual coupling during incongruent real and motor imagery tasks^6^, as confirmed in this work. This result might be explained, from a neurophysiological point of view, considering the brain areas involved in movement execution, motor imagery and proprioceptive illusion. Indeed, previous studies showed a consistent overlap of parietal and frontal brain regions activated during imagined, kinesthetically perceived and actually executed actions^21,33–37^. A similar prefrontal–parietal network (mostly involving right pre-SMA/CMA and bilateral PPC) was found to be responsible for the coupling effect in circle-line task, discriminating between congruent and incongruent conditions both in Real and MI protocols^6^. Therefore, the overlap between brain regions activated in these three conditions could explain the bimanual coupling here obtained in PI condition.

From a behavioral point of view, it has been demonstrated that the subjective perceptual content of illusory movements is very similar to that of the kinesthetic pictures that are generated internally during imagined movements^38–40^. Furthermore, previous investigations showed that the proprioceptive evoked sensation can combine with an imagined or an observed movement leading to behavioral and neurophysiological changes. Indeed, this combination generated movements that were the sum of the vectors of the sensations evoked by the two modalities^40,41^ and was responsible of inducing neuroplasticity through long-lasting changes in primary motor cortex (M1) activity^26,42^. Nevertheless, Proprioceptive Illusion and Motor Imagery remains qualitatively different, since the first is a bottom-up information process in human sensory-motor systems lacking of motor programming, whilst motor imagery can be considered as a top–down streams of information^41^. PI is therefore a peripheral simulation that is integrated at central level, and the cortical processes underlying the presence of movement illusion have already been demonstrated^22^.

Bilateral transfer effect of proprioceptive inputs has been shown also following passive movements over involuntary contralateral movements^43–45^, phenomenon that might be ascribed to the similarity among the brain regions activated by passive and actual movements^46^. However, it has been demonstrated that bimanual coupling during a circle–line task was unaffected by a controlateral passive movement^7^. A limitation of our study could consist in the absence of a direct comparison between passive movement and proprioceptive illusion of movement, since both are peripheral stimulation. However, we think that PI is more than just an afferent input coding an induced movement. In fact, during tendon vibration subjects usually refer a sensation of a movement internally generated, even if they are well aware of the experimental conditioning^23^. We suggest that since proprioceptive illusion evoked by vibration is not perceived as a mere peripheral stimulation, but as an internally generated action, it could elicit in subjects sense of agency (namely the subjective feeling of controlling one's own action, and through it, external events)^47,48^, as other proprioceptive illusions do, like rubber hand illusion^49,50^. It has been demonstrated that patients with schizophrenia refer sense of agency over the experimenter’s hand performing a drawn, and it results in the ovalization of the trajectory they are performing unimanually^12^. This showed that an interference is possible even in absence of motor planning if the subject perceives the agency of the movement.

With regard to cortical processing of proprioceptive illusion, the interhemispheric transfer of proprioceptive information has been investigated using a short-term limb immobilization paradigm. In a study based on this paradigm, we showed that limb non-use reduced the excitability of the contralateral M1 and decreased the interhemispheric inhibition onto the ipsilateral one, whilst the opposite effect occurred for the ipsilateral M1^28^. Following these findings, in a successive study, we demonstrated that hemispheric unbalance induced by limb non-use could be significantly reduced by the administration of proprioceptive inputs by means of muscle vibration during limb immobilization^29^. However, the obtained results were not able to disentangle whether this effect was due to the localized proprioceptive integration in the sensorimotor areas of the stimulated limb or due to an amount of proprioceptive information actually transferred to the non-stimulated hemisphere. In the present work, the results obtained using the bimanual coupling paradigm during proprioceptive illusion indicate the occurrence of a proprioceptive information interhemispheric transfer during muscle vibration.

The proprioceptive influence on bimanual coupling partially differed to that observed in Real and MI condition. Indeed, in PI during incongruent condition, ovalization appeared in Lines trajectories but not over Circle trajectories. One possibility to explain this difference is that when performing the circle-drawing task participants focused on this activity more than on the perceived illusory sensation because the circle-drawing task was more demanding. Also, the circle drawn is bigger than that of the lines and requires wider movements. Since tendon vibration illusion is also influenced by proprioceptive information from posture and trunk position^21,51–54^, this difference could justify the lower influence over the other hand. Vice versa, the line pattern might be easier to perceive, requiring less attentional resources and thus increasing attention devoted to the task performed with the contralateral hand. Indeed, other researchers have relied just on line-drawing rather than circle-drawing to study bimanual coupling^6,13,55^.

It is worth to mention that Della Gatta and colleagues found different effects over circle- and lines-trajectories while investigating whether collective goals induce bimanual coupling during a modified version of this task. In this work one actor had to draw sequential lines while observing another participant drawing sequential circles, and vice versa, stressing that the resulting design was a collective goal^5^. In this case, authors found an ovalization of the trajectory in the actor performing the line-drawing while observing the circle-drawing, but no significant effect over the circle-drawing, as in the present case.

Concerning movement velocity, we found a difference between the Real condition and the other two conditions (MI and PI) in both the line and circle drawings; subjects were slower when performing a bimanual task while imagining or perceiving the movement illusion. Our hypothesis is that in MI and PI conditions subjects were actually performing a dual task: one consisted in the real performance of the drawing and the second in a cognitive task, as it can be the imagination of a movement or the monitoring of the perceived illusion. This dual task requires an attentional switching between the performance of the left and the performance of the right hand. It is has been demonstrated in MI tasks that the attentional switching lead to an increase in the cognitive effort^56^, and thus a decrease in the speed, and also that this effect was greater in MI than in overt actions^57^. In our opinion, the slowing of the velocity in the PI condition is a demonstration of some level of interference between hemispheres, even in absence of an ovalization effect.

It may be argued that subjects were also imagining the movement during PI tasks in order to ease the perceived illusion. Nevertheless, the difference emerging between protocols in circles- and lines-drawing supports that the ovalization effect is strictly related to the evoked sensation in PI, and that subjects were actually perceiving the sensation emerging from the vibration, and not just imagining it.

The relevance of the present study is that we went beyond the previous observation on the bimanual coupling, showing that proprioceptive illusion could interfere with motor program of the contralateral hand, and be transferred to the contralateral hemisphere, influencing its activity. Indeed, we demonstrated that, in specific conditions, the proprioceptive information produced by muscle vibration could be sufficient to induce bimanual coupling. Also, we think that this finding will be useful in investigating the never explored role of proprioception in the phenomenon of 'cross education' training^58^.

## Materials and methods

### Participants

Fifteen healthy participants (8 females; age mean ± ES: 24.73 ± 1.53 years) were recruited for the experiment. All subjects were right-handed, according to the Edinburgh Handedness Inventory^59^. None of the subjects had history of orthopaedic or neurological illness or any motor or sensory deficit related to upper limb. The experimental protocol was approved by the ethics committee of the University of Genoa (Comitato Etico per la Ricerca di Ateneo—CERA, N. 2020/18), and was carried out in agreement with legal requirements and international norms (Declaration of Helsinki, 1964). All subjects gave informed consent for participation in the study.

### Experimental setup

Participants were seated on a chair in front of a table on which an Apple iPad Pro (12.9″) was placed, positioned to the left of the participant’s sagittal midline. Participants handled an Apple Pencil with their left hand in order to draw on the iPad and were blindfolded for the entire duration of the experiment.

Subjects underwent three versions of the Circle-Line paradigm adapted for the required behavioural task: Real (RE), Motor Imagery (MI) and Proprioceptive Illusion (PI). The behavioural manipulations were performed over the right wrist/hand (conditioned hand), whilst the left hand always drew a real figure (target hand). The collected drawing data were processed to evaluate possible effects induced by the experimental paradigm.

The tasks consisted of drawing with the left hand continuous vertical lines or circles: in the line task, reciprocal lines along the y-dimension were reproduced for 50 s; in the circle task, circles were traced, without any indication about the direction of motion, for 50 s. Data were collected with a custom-made software from the iPad.

### Experimental design

Each subject performed, in a randomized order, three experimental protocols: Real protocol, MI protocol and PI protocol. Each protocol was performed in a Congruent and Incongruent condition, for both the line and the circle task, and repeated six time, for a total of seventy-two trials. For the left hand, and for the right hand in the Real protocol, subjects were asked to perform ecological, self-paced movements rather than externally imposing a fixed movement frequency. Subjects were requested to perform the task moving mostly their wrist and fingers, but keeping the arm raised from the table, in order to avoid a pivot effect from the elbow.

### Real protocol

In the Real protocol, subjects were asked to draw lines or circles with both hands, the left with the Apple Pencil on the iPad, the right with a pen on a paper sheet.

In particular, during the Real congruent protocol, subjects simultaneously drew vertical lines with both hands (RE_Line-Congruent) or circles with both hands (RE_Circle-Congruent). In the Real incongruent protocol, participants simultaneously drew lines with the target hand and circles with the conditioned hand (RE_Line-Incongruent) or vice versa (RE_Circle-Incongruent).

### Motor imagery protocol

In the MI protocol, subjects were asked to kinaesthetically imagine drawing a figure with their right hand. To ease the task, subjects held a pen in their right still hand with the pen point placed over the paper sheet. At the same time, they drew with their left hand on the iPad. In particular, in the MI congruent protocol, subjects imagined to draw lines/circles with their conditioned hand and, simultaneously, they drew lines/circles with their target hand (MI_Line-Congruent and MI_Circle-Congruent, respectively). Conversely, during the MI incongruent protocol, subjects imagined to draw circles with their conditioned hand and, simultaneously, they drew lines with their target hand (MI_Line-Incongruent) or vice versa (MI_Circle-Incongruent).

### Proprioceptive illusion protocol

In the PI protocol subjects placed the wrist of the right hand in a custom-made apparatus with four electromechanical vibrators. They were asked to keep the hand in a natural position like they were writing, with their thumb pointing up.

**Figure 4:**
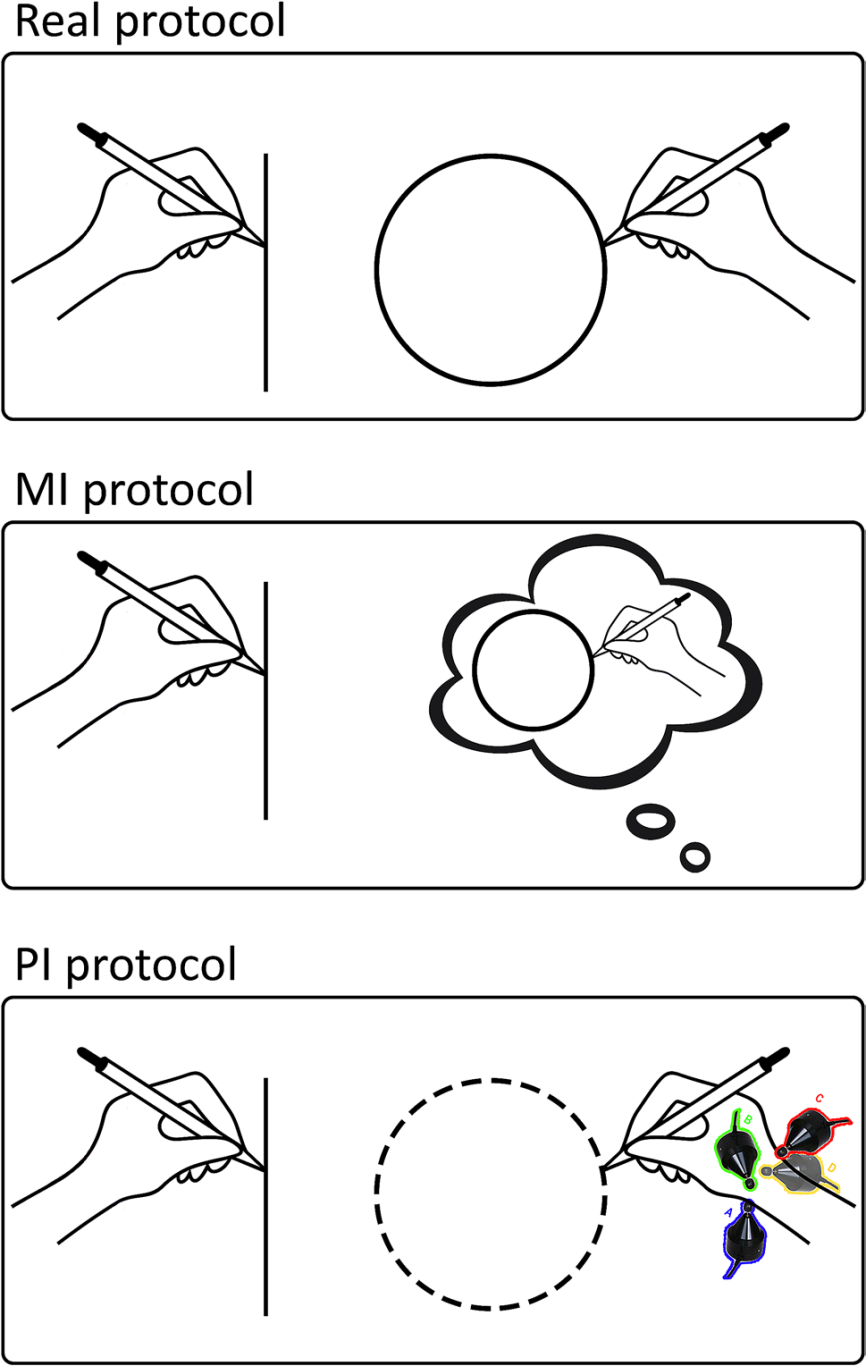
Experimental paradigm. Images show a rapresentative Line-incongruent condition in the Real, Motor Imagery—MI and Proprioceptive Illusion—PI protocols. The left hand actually performed continuous line drawings. In the Real protocol (top) the subject has to actually perform repetitive drawings of circles. In the MI protocol (middle) the subject has to kinaesthetically imagine to perform repetitive drawings of circles. In the PI protocol (bottom) four stimulator were orientated to stimulate 4 tendons of the subject’s wrist. The dotted line represent the stimulation pattern of repetitive circles. Figure were created with Adobe Photoshop (version CC 2015. https://www.adobe.com/).

The head of the four vibrators was applied perpendicularly to the tendons of the dorsal, volar, radial, and ulnar muscles at the level of the wrist joint, as shown in Fig. 4. The vibration frequency to be administered was controlled by a custom-made software program and the amplitude was set at 1 mm peak-to-peak.

Different vibrating patterns were administered to wrist muscle tendons to induce illusory hand movements of drawing circles or lines. The proprioceptive illusion of drawing sequential vertical lines was induced by inducing alternate “flexion–adduction”—“extension–abduction” sensations. Such sensations were evoked by the simultaneous stimulation of the dorsal-ulnar tendons and volar-radial tendons. The stimulation for each portion of movement was a constant vibration set at 80 Hz for 4 s for a total of 50 s. The kinaesthetic illusion of drawing sequential circles was induced by activating consequential couples of adjacent vibrators. The stimulation for each portion of movement was an increasing and decreasing vibration from 50 to 80 Hz for a total of 50 s. Vibration patterns are reproduction of the ones described in Roll and Gilhodes^60^.

For each subject, the position over the wrist and the orientation of the stimulating head were established for each vibrator singularly by asking participant to report the illusory movement sensation in term of movement direction and vividness of illusion. Once the experimenter found the best stimulation position and orientation for each stimulator, each participant familiarized with the stimulating protocol, in order to verify the expected illusory sensation. Stimulation patterns related to circles and lines were administered. During familiarization phase, participants were asked to reproduce with their controlateral (left) hand the perceived illusory movement after the 50 s-stimulation pattern. In this phase, subjects were also asked to report the subjective experience of the proprioceptive illusion in order to exclude any difference in the level of proprioceptive illusion between the two stimulation patterns. To this aim, a Likert scale from 0 to 10 concerning the vividness of the movement illusion (i.e., the clarity of the illusion in comparison with an actual drawing) was administered. In particular, 0 corresponded to “no illusion” and 10 corresponded to “strong illusion of real movement”. No previous information were given to participants concerning the orientation, speed and direction of the movement they would have perceived during the proprioceptive stimulation.

During the PI congruent protocol, subjects perceived the movement illusion of drawing a line with the conditioned hand and simultaneously drew vertical lines with the target hand (PI_Line-Congruent), or they perceived to draw a circle with the conditioned hand while drawing a circle with the target hand (PI_Circle-Congruent). Conversely, in the PI incongruent protocol, subjects perceived the movement illusion of drawing circles with the conditioned hand and simultaneously they drew lines with the target hand (PI_Line-Incongruent) or, vice versa, the illusion of drawing lines was administered in the conditioned hand and circles were drawn with the target hand (PI_Circle-Incongruent).

### Data and statistical analysis

#### Data analysis

Data collected from subject’s left hand and recorded by the iPad were analysed through a custom-made MatLab software, designed ad-hoc for this experiment. x and y coordinates of the written trace were low-pass filtered at 5 Hz using a 2nd order Butterworth filter. Then, through a semi-automated procedure, in each trial the written trace was segmented in circles or reciprocal lines that composed it on the basis of the velocity profile. For each segmented circle/reciprocal lines two variables were computed: the ovalization index (OI) and the mean velocity (MV).

The ovalization index (OI) allowed quantifying the deviation from a perfect line or a perfect circle. OI was computed as follows. We computed the velocity profile on y dimensions. Then, four points were automatically identified: the first zero cross, the positive peak velocity, the second zero cross, and peak negative velocity^4^. The x and y position coordinates at each of these velocity landmarks were used to compute distances joining the points at the zero crossings and the points at the peak velocities. The ratio of these two distances was computed with the larger of the two numbers as the denominator. Thus, the index of ovalization was within the range 0 ÷ 1, with 0 indicates perfect reciprocal lines and 1 indicates a perfect circle.

The mean velocity (MV) was obtained for each circle/reciprocal lines by averaging the module of the velocity profile (taking into account both x and y component). By means of MV we explored the variation in the temporal aspects of the tasks.

### Statistical analysis

According to Shapiro–Wilk test, all the experimental data were normally distributed.

Since the illusory sensation was a prerequisite for the experiment, a paired t-test as preliminary analysis was adopted to compare vividness rate recorded during the familiarization with the proprioceptive illusion.

OI and MV of each circle/reciprocal line were averaged in each trial. Then, the representative value for each subject in each experimental condition was obtained as the average of the mean value across the six trials.

Mean values of OI and MV were subjected to two repeated‐measures ANOVA with PROTOCOL (3 levels: RE, MI, PI) and CONGRUENCY (2 levels: Congruent, Incongruent) as within-subjects factors, separately for Lines and Circles. Significant interactions in the ANOVA were followed by post hoc Newman–Keuls tests. Moreover, to further explore the ovalization of the PI protocol only, we adopted a paired t-tests between its congruent and incongruent conditions (adjusted p value for multiple comparison = p = 0.05/3 = 0.017).
